# Supplementary figures and images for: Comparison of Read Mapping and Variant Calling Tools for the Analysis of Plant NGS Data
Source: Plants (Basel). 2020 Apr 2;9(4):439. doi: 10.3390/plants9040439 (PMC7238416; doi:10.3390/plants9040439)

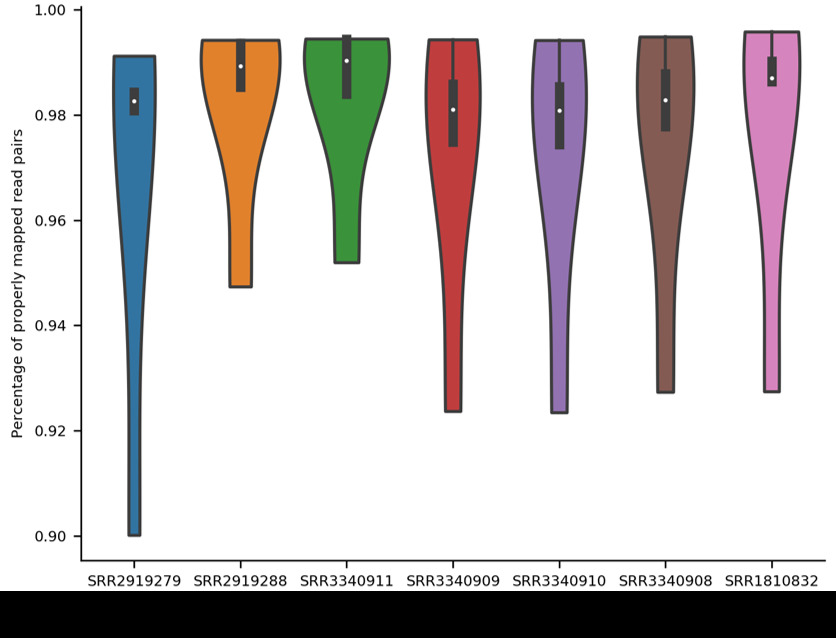

Supplement: Supplementary file 1 [file plants-09-00439-s001.zip › Supplementary_Material/Figure S1.jpg]

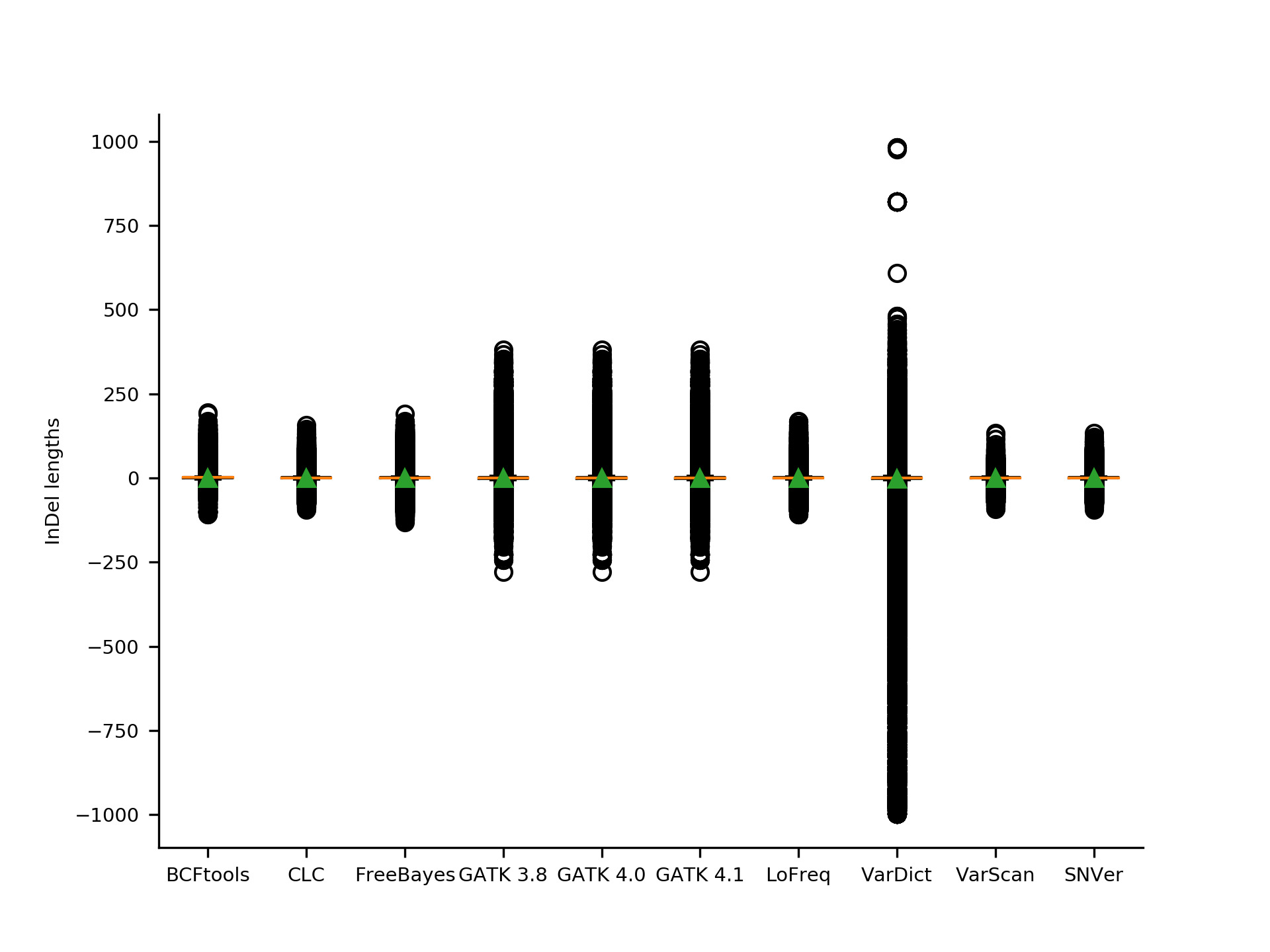

Supplement: Supplementary file 1 [file plants-09-00439-s001.zip › Supplementary_Material/Figure S2.jpg]

Specificity

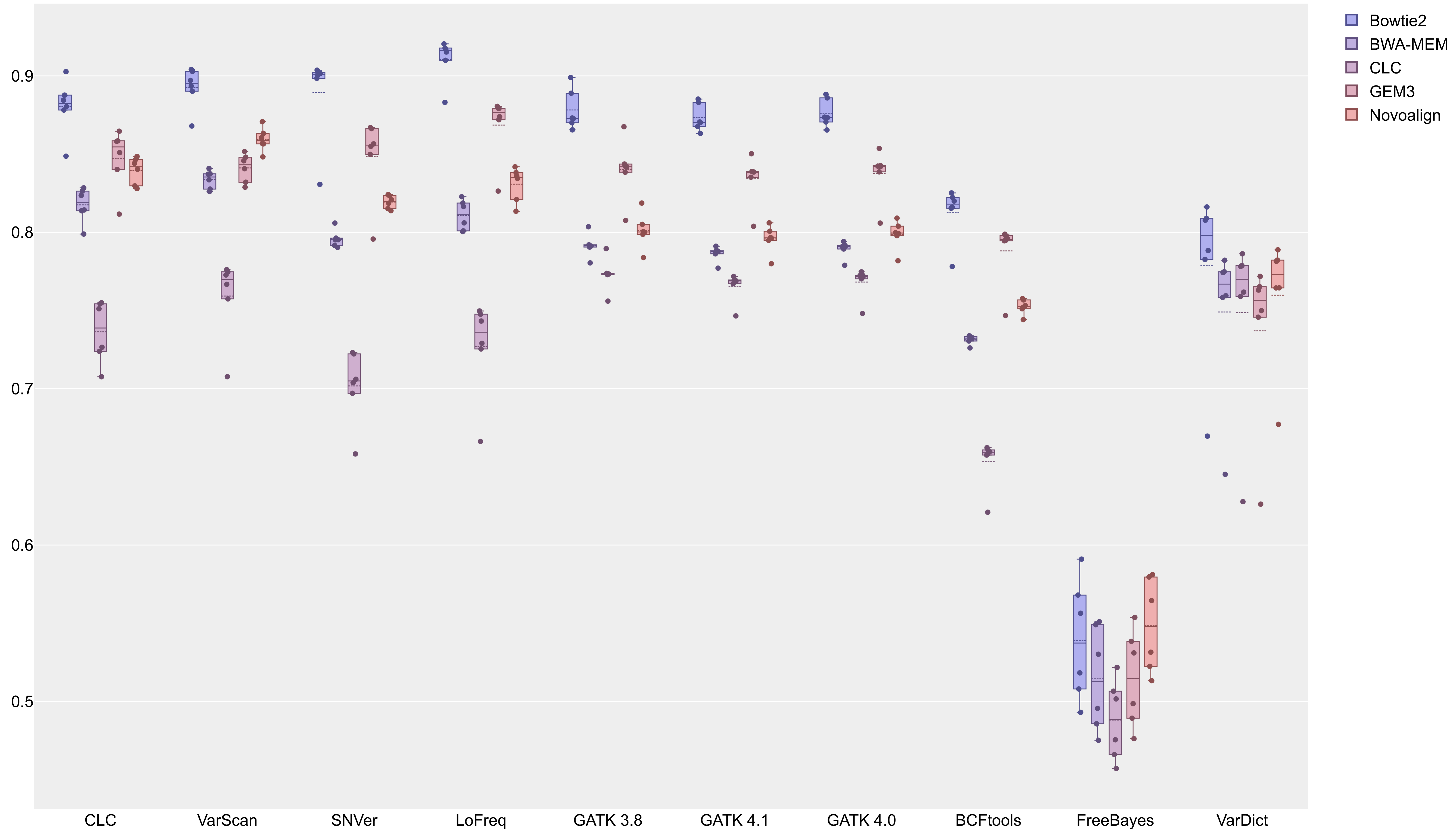

Supplement: Supplementary file 1 [file plants-09-00439-s001.zip › Supplementary_Material/Figure S3.pdf]

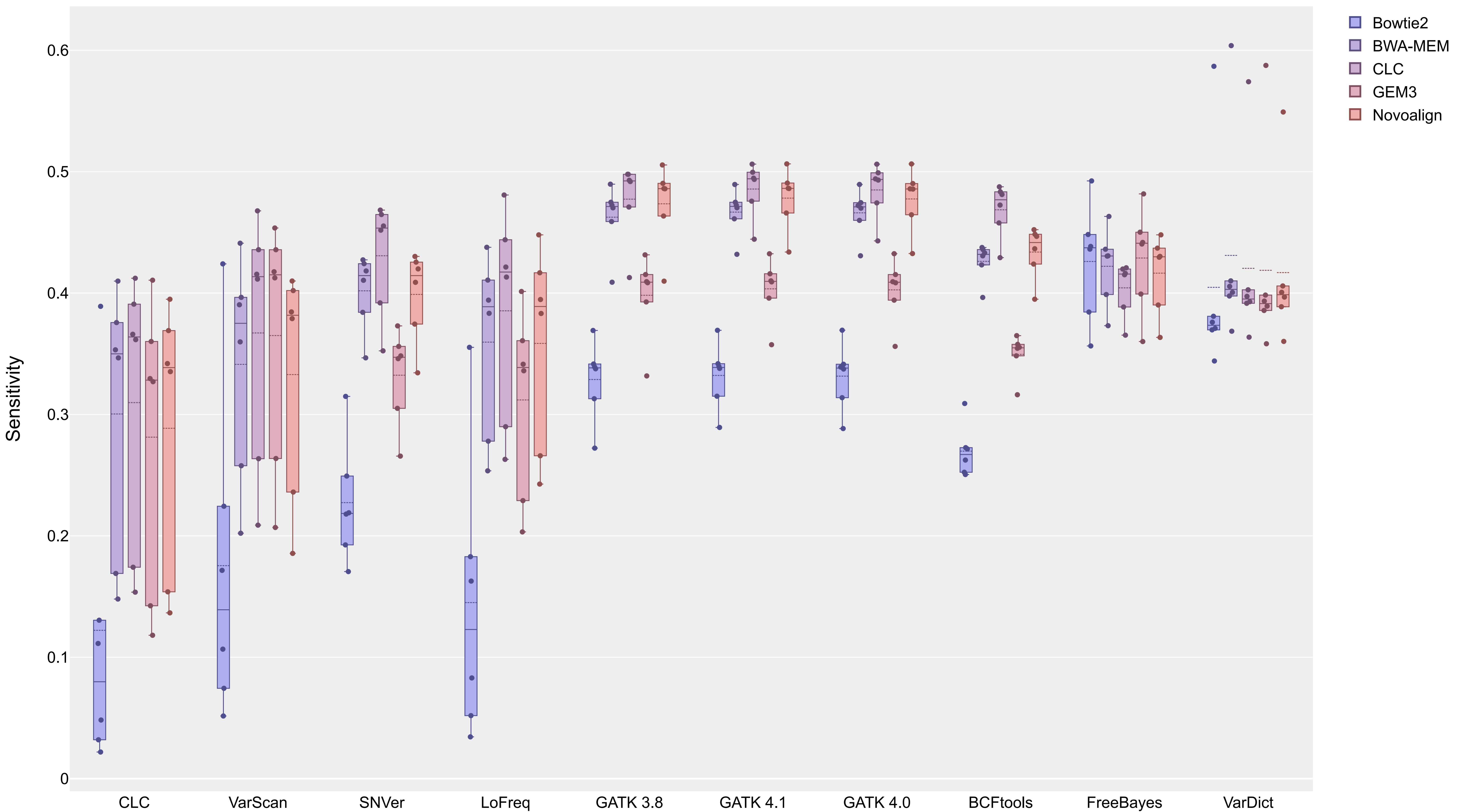

Supplement: Supplementary file 1 [file plants-09-00439-s001.zip › Supplementary_Material/Figure S4.pdf]

F1 score

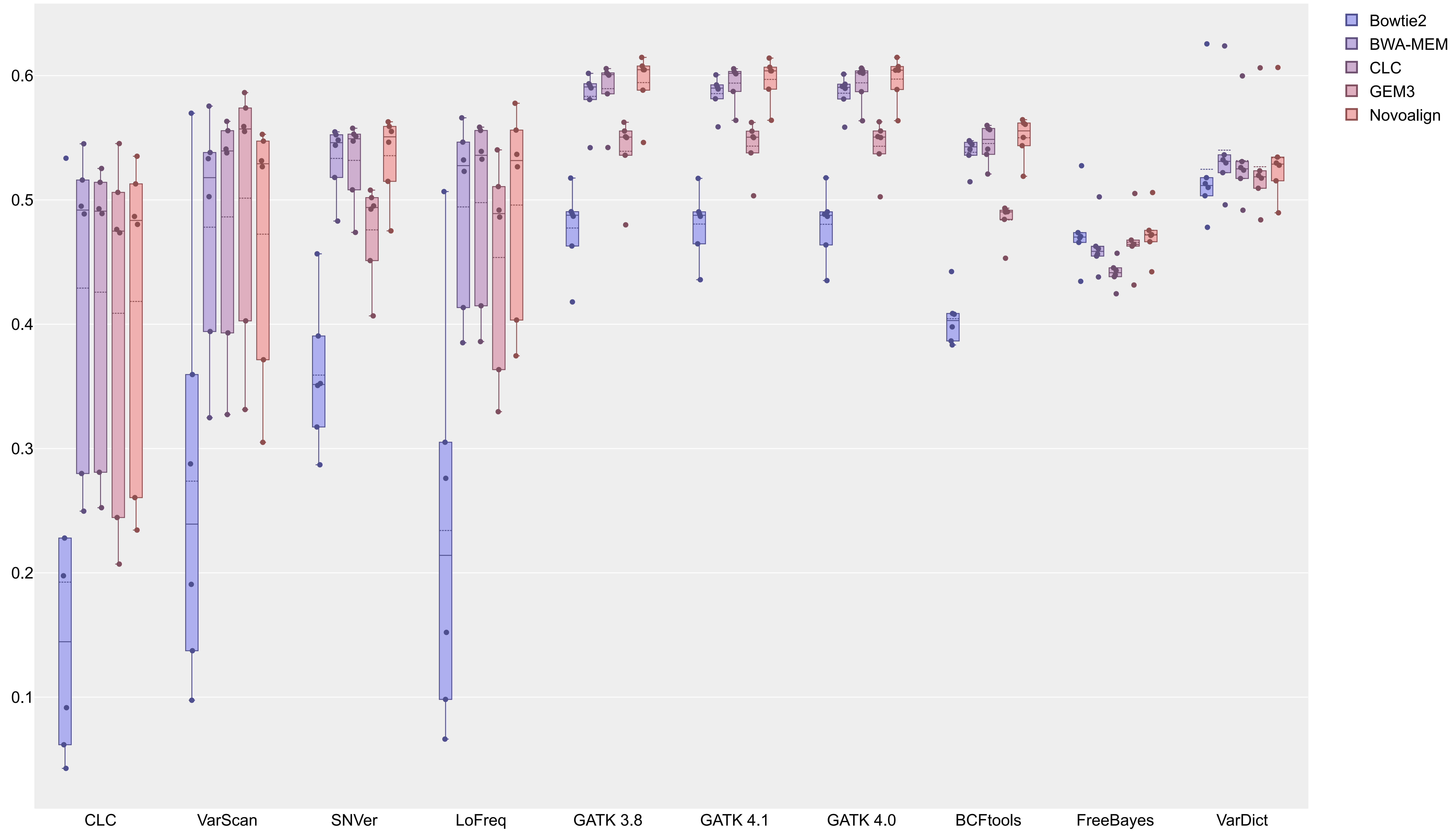

Supplement: Supplementary file 1 [file plants-09-00439-s001.zip › Supplementary_Material/Figure S5.pdf]

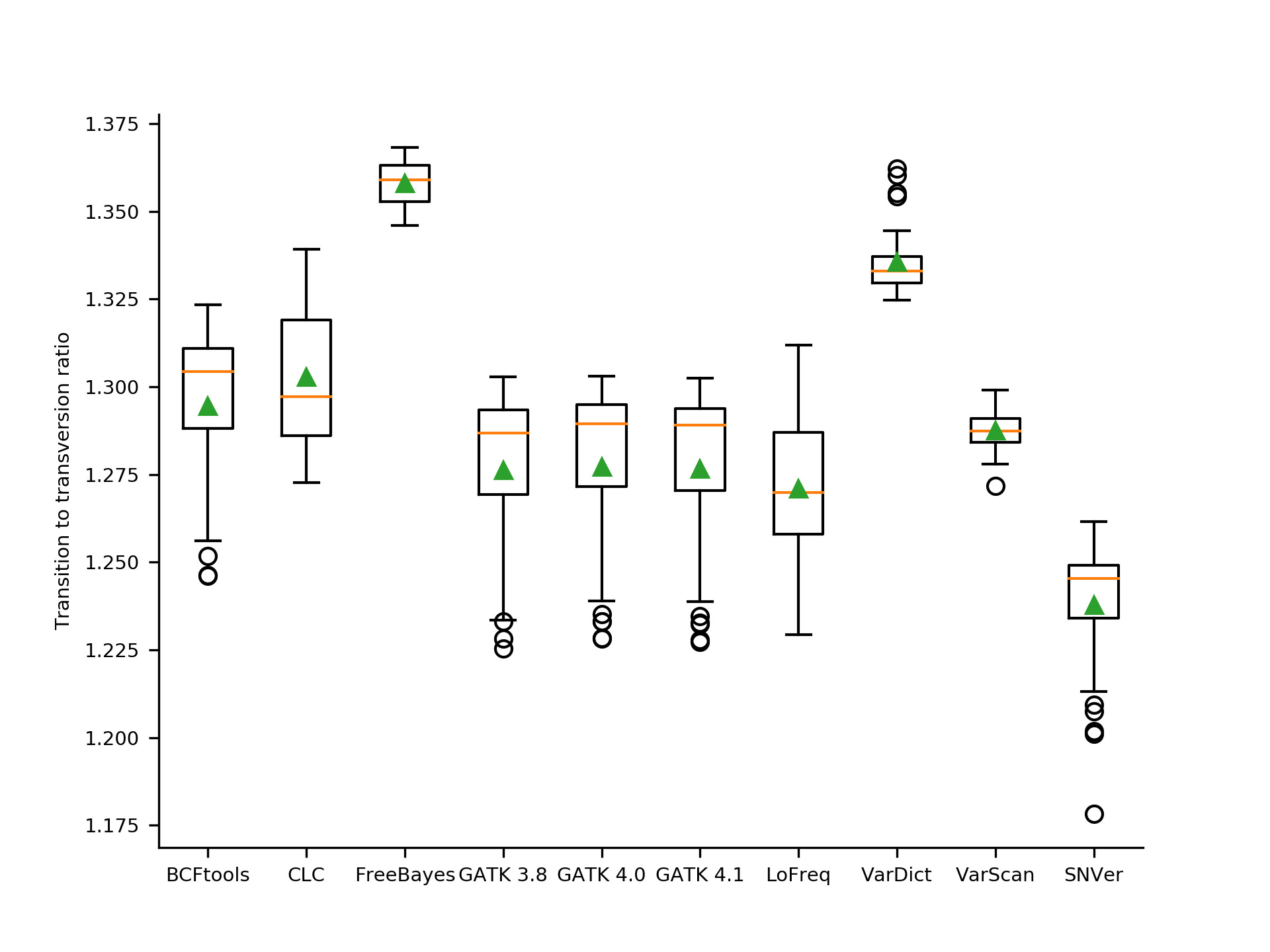

Supplement: Supplementary file 1 [file plants-09-00439-s001.zip › Supplementary_Material/Figure S6.jpg]
